# Feeding Laying Ducks *Eucommia ulmoides oliv.* Leaves Increases the n-3 Fatty Acids Content and Decreases the n-6: n-3 PUFA Ratio in Egg Yolk without Affecting Laying Performance or Egg Quality

**DOI:** 10.3390/foods12020287

**Published:** 2023-01-08

**Authors:** Yulong Feng, Guotao Dai, Xue Han, Meijuan Li, Degang Zhao, Jiahai Wu, Yongbao Wu, Zhiguo Wen

**Affiliations:** 1 Institute of Animal Husbandry and Veterinary Medicine, Guizhou Academy of Agricultural Sciences, Guiyang 550005, China; 2 Plant Conservation Technology Center, Guizhou Key Laboratory of Agricultural Biotechnology, Guizhou Academy of Agricultural Sciences, Guiyang 550006, China; 3 Institute of Feed Research of Chinese Academy of Agricultural Sciences, Key Laboratory of Feed Biotechnology of Ministry of Agriculture, Beijing 100081, China

**Keywords:** n-3 polyunsaturated fatty acids, polyunsaturated fatty acids, *Eucommia ulmoides oliv.*, cholesterol, laying ducks, performance

## Abstract

The objective was to determine the effects of supplementing duck diets with *Eucommia ulmoides oliv.* leaf powder (EUL). Laying ducks (*n* = 480) were randomly allocated into 4 experimental treatments and fed diets containing 0, 1, 2, or 4% EUL. Dietary inclusion of EUL had no effect (*p* > 0.05) on laying performance or egg quality, but linearly increased (*p* < 0.05) total plasma protein, globulin, and HDL-C concentrations with concurrent reductions (*p* < 0.05) in plasma concentrations of cholesterol and LDL-C. Eggs laid by ducks receiving EUL had yolks with linearly higher phenolic concentrations (*p* < 0.05) but lower cholesterol concentrations (*p* < 0.05). EUL supplementation in duck diets significantly reduced n-6: n-3 PUFA ratio by enriching n-3 fatty acids in yolks (*p* < 0.05) with no changes in n-6 PUFA (*p* >0.05).

## 1. Introduction

Eggs are an excellent source of essential amino acids, essential lipids, fat-soluble vitamins, choline, and carotenoids [1]. Furthermore, eggs are relatively rich in fatty acids and bioactive compounds (e.g., phenols, carotenoids, and flavonoids) which can be enhanced through dietary intervention [2,3,4,5]. The type and ratios of fatty acids, especially polyunsaturated fatty acids (PUFA) of the n-3 and n-6 families and the n-6: n-3 PUFA ratio, are used to assess the nutritional value of an egg [6]. It is well known that n-3 PUFA, consisting of α-linolenic acid (ALA, C18:3 n-3), docosahexaenoic acid (DHA, C22:6 n-3), docosapentaenoic acid (DPA, C22:5 n-3), and eicosapentaenoic acid (EPA, C20:5 n-3), have beneficial roles in preventing cardiovascular disease (CVD), plus inflammatory and autoimmune diseases [7,8]. In contrast, eating excessive amounts of n-6 PUFA or diet with a very high n-6: n-3 ratio increases the risks of CVD and inflammation [9]. Therefore, both a balanced n-6: n-3 PUFA ratio and increased n-3 PUFA intake are important in preventing CVD and other metabolic diseases [9,10]. Poultry scientists have been seeking ways to enhance yolks’ n-3 PUFA contents and to maintain a balanced n-6: n-3 PUFA ratio in yolks.

The fatty acid composition of egg yolk can be modulated by diet [2,11,12]. There are 2 dietary intervention strategies for the enrichment of eggs with lipids of biological value. One method of increasing n-3 PUFA content in egg yolk is feeding fish oils and marine algae rich in long-chain n-3 PUFA [3,12,13,14], although this may cause a fishy odor or taste [15,16]. Alternatively, n-3 PUFA content was increased by supplementing diets of laying poultry with natural plants rich in ALA, e.g., linseed, rubber seed oil, herbs, or various vegetable oils [2,17,18,19], albeit with a lower enrichment efficiency compared to fish oil [20]. One plant of considerable interest was *Eucommia ulmoides oliv.*, a single genus of the Eucommiaceae family, due to its high ALA content and health-promoting properties [21,22].

*Eucommia ulmoides oliv.*, an important and valuable traditional Chinese medicine, has been widely grown in the south of China for medicinal purposes for decades. The planting area in China is about 33 million, accounting for over 99% of the total resources in the world [22]. Traditionally, only the bark has been used as medicine, whereas leaves were usually not fully used, despite having almost the same bioactive compounds as bark [22]. Santoso et al. [23] reported that *Eucommia ulmoides oliv.* leaves contained 14.3%, 6.7%, and 8.9% of crude protein, fat, and crude fiber, respectively. Xing et al. [22] reviewed the chemical constituents in bark and leaves and found that 132 chemical compounds were identified, including phenols, flavonoids, polysaccharides, gutta-percha, and fatty acids. Zhang et al. [21] found that the major PUFA in seed oil of *Eucommia ulmoides oliv.* were ALA and linolelaidic acid, accounting for 56.50% and 12.65% of total fatty acids, respectively. Therefore, there is a need to explore ways to better utilize this plant. Positive effects of consuming *Eucommia ulmoides oliv.* leaf powder or extract supplementation on animal health or performance were reported in model animals [24,25], but there are limited studies on poultry [23,26,27]. In summary, *Eucommia ulmoides oliv.* leaf powder or extract had no advantageous effect on growth or laying performance. To our best knowledge, there was no systematic study about the effects of *Eucommia ulmoides oliv.* on the fatty acid composition of egg yolk on laying poultry. In the present study, we hypothesized that supplementing duck diets with EUL improves the yolk fatty acid profile and the health-promoting values of eggs, due to their high contents of ALA and bioactive compounds.

## 2. Materials and Methods

### 2.1. Birds, Diets, and Experimental Design

A dose-response experiment with 4 dietary treatments was conducted in a completely randomized design. Four hundred and eighty old laying ducks (Sansui Sheldrake duck) aged 32 wk with similar laying rates and initial body weight were randomly divided into 4 groups of 8 replicates with 12 birds. Birds were raised in 4-level battery cages under a controlled ventilation and lighting house, providing 16 h of light and 8 h of dark. To minimize the cage level effects, 4 vertically successive cages with one diet were arranged as a replicate. Each cage (width 60 cm, length 60 cm, and height 40 cm) included 3 birds. The ambient temperature was maintained at 24 ± 2 °C throughout the experiment. Diets in pellet form and water were provided ad libitum. The feeding period was 8 weeks, including a 1-week adaption feeding period.

Corn-soybean meal basal diets (Table 1) were supplemented with 0, 1, 2, or 4% of *Eucommia ulmoides oliv.* leaf powder (EUL) at the expense of wheat bran and rice hull powder and formulated to meet the nutrient requirements of laying sheldrake ducks [28]. *Eucommia ulmoides oliv.* were planted in Guizhou province of China. Fresh leaves were collected in July 2021, and treated with steam (100–110 °C) for 10 s, dried, and pulverized to a fine powder. Chemical components of the EUL used in this study are shown in Table S1.

### 2.2. Data Collection and Sample Preparation

Laying performance was recorded daily based on a replicate, including egg weight, egg number, shell-less eggs, and cracked eggs. Feed intake was recorded weekly on a replicate cage basis. In retrospect, egg production, total egg mass, daily egg mass, average egg weight, uncracked shell egg rate, and daily feed intake were calculated for the entire experiment. Feed conversion ratio was calculated and expressed as per kilogram of feed consumed per kilogram of eggs produced for the entire experimental phase.

On the last day of the experiment, 16 randomly selected eggs from each group (2 eggs per replicate) were collected for analysis of egg quality parameters, including egg yolk weight, egg yolk percentage, egg shape index, albumen height, eggshell strength, eggshell thickness, Haugh units (HU) and egg yolk color. Furthermore, egg yolks were collected and freeze-dried for further analyses of dry matter, total lipid, total protein, total cholesterol, total triglyceride, and fatty acids composition. Meanwhile, blood samples, ~2 mL, were collected into heparin sodium-anticoagulant tubes from brachial vein of 2 ducks per replicate and centrifuged at 3000 RPM for 10 min, and plasma was harvested and stored at −20 °C pending biochemical analyses.

### 2.3. Sample Analyses

Egg samples collected above were weighed individually, and egg shape index was calculated by measuring vertical length and width using a vernier caliper, as described by Aydin et al. [18]. The indices of eggshell strength and eggshell thickness were measured using Egg Force Reader and Eggshell thickness (ORKA Food Technology Ltd., Ramat Hasharon, Israel), respectively. Albumen height, HU, and yolk color were measured using Egg Analyzer (ORKA Food Technology Ltd., Ramat Hasharon, Israel). After measuring these indices, yolks were collected and weighed before and after being dried in a freeze dryer, and yolk percentage was expressed as the ratio of fresh yolk weight to egg weight. Meanwhile, the dry matter of the yolk was calculated. Dried yolk samples from a replicate were pooled and mixed homogeneously for further analyses.

Total protein content (6.25 × crude nitrogen) of yolk was measured by determining crude nitrogen content using the Kjeldahl method. Yolk lipids were extracted in chloroform-methanol (2:1), as described by Folch et al. [29]. Total triglyceride and total cholesterol contents of the egg yolk were determined using an ultraviolet spectrophotometer and commercial kits (Nanjing Jiancheng Bioengineering Institute, Nanjing, China) as described by Zhang et al. [30]. Total flavonoids and total phenolic contents of yolk were determined spectrophotometrically according to Muhammad et al. [31] using commercial kits (Nanjing Jiancheng Bioengineering Institute, Nanjing, China), following the manufacturer’s instructions.

Plasma concentrations of alanine aminotransferase (ALT), aspartate aminotransferase (AST), total bile acid (TBA), total protein, albumin, glucose, uric acid, cholesterol, triglyceride, high-density lipoprotein cholesterin (HDL-C), and low-density lipoprotein cholesterin (LDL-C) were evaluated using an automatic analyzer (Hitachi 7080, Tokyo, Japan) and commercial kits (Maccura, Sichuan, China). Plasma globulin concentrations were calculated by the difference between plasma concentrations of total protein and albumin.

Freeze-dried yolk samples were pulverized and passed through a 40-mesh sieve for analyses of fatty acids. The fatty acid methyl esters of the total lipids were prepared according to the procedure described by Fredriksson et al. [3]. Both quantitative and qualitative analyses were performed using an Agilent 7890B gas chromatograph system coupled with an Agilent 5977B mass spectrometer (Agilent Technologies, Santa Clara, CA, USA). The system used a DB-FastFAME capillary column (90 m × 250 μm × 0.25 μm). An aliquot (1 μL) of the analyte was injected in split mode (5:1). Helium was used as the carrier gas, the front inlet purge flow was 3 mL/min, and the gas flow rate through the column was 46 psi with constant pressure. The initial temperature of column was kept at 75 °C and held for 1min, subsequently increased to 200 °C at the rate of 50 °C/min and held for 15min, then to 210 °C at the rate of 2 °C/min and held for 1min, finally to 230 °C at the rate of 10 °C/min and held for 16.5 min. Temperatures of front injection, transfer line, quad, and ion source were 240, 240, 230, and 150 °C, respectively. Electron energy was −70 eV in electron impact mode. Mass spectrometry data were acquired in Scan/SIM mode with the *m*/*z* range of 33–400 after a solvent delay of 7 min. Identification of fatty acids in samples was conducted using a combination of retention times and mass spectral characteristics. Fatty acids were quantified by comparing the areas of the peaks with those of known standard substances (Anpel Laboratory Technologies Inc., Shanghai, China).

### 2.4. Statistical Analyses

Data are presented as mean values with their pooled standard errors and analyzed by one-way ANOVA using SAS software (SAS Institue, Cary, NC, USA, 2003) **[32]**. Replicate was designated the experimental unit for analysis. When dietary treatment was significant (*p* < 0.05), means were compared using Tukey’s studentized range test procedure of SAS software (SAS Institue, Cary, NC, USA, 2003) [32]. For all analyses, a probability level of *p* < 0.05 was considered to be statistically significant.

## 3. Results

### 3.1. Laying Production Performance

The effect of dietary EUL levels on laying production was shown in Table 2. There were no significant effects of dietary EUL content on total egg mass, egg production, average egg weight, daily feed intake, feed conversion ratio, daily egg mass, and uncracked shell egg rate (*p* > 0.05).

### 3.2. Plasma Biochemical Indices

Increasing EUL supplementation linearly increased (*p* < 0.05) plasma total protein, globulin, and HDLC concentrations with a concomitant decrease (*p* < 0.05) in cholesterol and LDLC concentrations (Table 3). However, there were no differences (*p* > 0.05) in ALT, AST, TBA, uric acid, albumin, glucose, or triglyceride.

### 3.3. Egg Quality

There were no differences (*p* > 0.05) among treatments for egg weight, egg yolk weight, egg yolk percentage, egg shape index, eggshell thickness, albumen height, egg yolk color, HU, or eggshell strength at week 6 (Table 4).

### 3.4. Yolk Chemical Composition

Ducks fed diets supplemented with EUL had a lower (*p* < 0.05) and relatively constant cholesterol concentration in egg yolk than ducks receiving basal diets (Table 5). Total phenolic contents in egg yolk significantly increased with the increasing dietary level of EUL (*p* < 0.05). Compared to the basal diet group, the total phenolic contents in the 4% EUL group were improved by 0.42 mg/g (3.24 mg/g vs 3.66 mg/g). However, dry matter, crude protein, total lipid, total flavonoid contents, and total triglyceride in yolk were not different among treatments (*p* > 0.05).

### 3.5. Yolk Fatty Acids Composition

In total, 23 fatty acids were identified from all egg yolk samples in this study and categorized into saturated fatty acids (SFA), monounsaturated fatty acids (MUFA), and polyunsaturated fatty acids (PUFA) containing n-3 PUFA and n-6 PUFA (Table 6). The main fatty acids in duck egg yolk were C18:1 n-9 and C16:0. Increasing dietary EUL content reduced total SFA and increased total MUFA (*p* < 0.05 for each), but did not significantly affect the proportion of total PUFA. Specifically, there were significant linear decreases in C16:0 and C18:0 (*p* < 0.05) as dietary EUL levels increased. Among MUFA identified in this study, only C18:1 n-9 was linearly increased (*p* < 0.05) in response to dietary EUL levels.

The n-3 PUFA content, calculated as the sum of ALA, DPA, EPA, and DHA, was linearly increased with the increase of dietary EUL supplementation (*p* < 0.05), whereas there was no significant change in n-6 PUFA content (*p* > 0.05), consisting of C18:2 n-6 (linoleic acid, LA), C18:3 n-6 (γ-linolenic Acid, GLA), C22:2 n-6, C20:3 n-6, C20:4 n-6 (arachidonic acid, AA), C22:4 n-6, and C22:5 n-6. Among n-3 PUFA analyzed in yolks, ALA, DPA and DHA were linearly increased, with a simultaneous decrease in EPA (*p* < 0.05), as the EUL percentage increased. As the ratio of n-6 to n-3 PUFA and that of PUFA to SFA were important nutritional indicators for egg yolk, we next investigated whether dietary EUL supplementation improved these ratios. As expected, the n-6: n-3 PUFA ratio and PUFA: SFA ratio were decreased and increased linearly with increasing dietary EUL content (*p* < 0.05), respectively.

## 4. Discussion

There are reports on the benefits of EUL as a medical herb, e.g., improving hyperlipidemia [33] and maintaining hepatic health [25,34]. Therefore, we sought to evaluate the potential benefits of feeding EUL to laying ducks. As expected, feeding EUL did not elevate plasma ALT or AST enzyme activity, used as indicators of cell/liver damage in birds [35] and indicating that dietary EUL maintained liver health. Furthermore, increases in plasma total protein and globulin indicated dietary EUL may have a protective effect on health status [36]. In addition, diets supplemented with EUL decreased plasma LDL-C and cholesterol concentrations but increased HDL-C concentration supported the earlier conclusions that EUL had a beneficial role in regulating hyperlipidemia [24,33]. This might also explain the lower yolk cholesterol content, primarily synthesized in the liver and transported in plasma to the ovary [37]. Therefore, 1 to 4% EUL enhanced lipid metabolism and maintained health in laying ducks.

In this study, the overall production of laying ducks was not altered by the inclusion of EUL (up to 4%) in their diets. Similarly, hens fed 2.5% EUL had no significant alterations in laying performance, including egg production, egg weight, egg mass, feed intake, or feed conversion ratio [26]. It was reported that EUL extract (up to 500 mg/kg) did not improve the production performance of laying hens, except for a slight enhancement of laying rate [27], whereas inclusion of EUL in the diet of broilers had no significant effect on body weight gain, feed intake, or feed conversion ratio [23]. Therefore, our results supported the conclusion that EUL supplementation had no beneficial effects on poultry growth and production [26].

Egg-quality characteristics, including egg shape, egg yolk percentage, yolk color, and shell thickness, are important indicators for assessing the effects of any dietary interventions and influence egg consumers directly and indirectly. Under the present study conditions, there were no indications that EUL supplementation altered egg quality. To date, there are few reports regarding the effects of EUL or extract on egg quality. It was noted [27] that dietary supplementation of EUL extract had no significant effect on yolk color, egg shape index, shell strength, shell thickness, egg albumen height, HU, and yolk percentage in laying hens. Furthermore, in another report [26] supplemental EUL in hen diets did not significantly alter HU. Therefore, our findings were consistent with previous reports.

Dietary supplementation of herbal plants in laying poultry might enrich egg yolks with bioactive compounds from plants, e.g., essential oils, flavonoids, phenols, and carotenoids [2,3,5]. The EUL used in this study contained 72.36 mg/g and 13.41 mg/g of total phenols and total flavonoids, respectively; therefore, we speculated that these bioactive compounds would accumulate in egg yolk. As expected, there was a significant increase for yolk phenols in a dose-dependent fashion, but no significant effect on flavonoids in the yolk (despite a numerical increase).

In addition to no significant changes in laying production performance and egg quality among treatments, the linear response of egg fatty acids content to increasing dietary EUL suggested that those experimental diets were appropriate for evaluating the EUL application effect in laying ducks. Both LA and ALA are essential fatty acids for poultry, which must be provided by the diets because these are indispensable for health yet cannot be synthesized [38]. In the liver, the same desaturase (Δ-6 and Δ-5 desaturase) and elongase enzymes catalyze LA and ALA into n-6 and n-3 PUFA, respectively. First, ALA was converted to EPA, then elongated into DPA, and finally synthesized into DHA. Similarly, LA was converted step by step into LA derivatives, including C18:3 n-6, C20:3 n-6, C20:4 n-6, C22:4 n-6, and C22:5 n-6. As we all know, n-3 PUFA played important roles in cardiovascular disease prevention, nervous system development, anti-inflammation, and antioxidants. Foods enriched with functional ingredients, such as n-3 PUFA and flavonoids, have become popular among consumers. In this study, there was no significant effect of ELU supplementation on LA derivatives, whereas there were major effects on n-3 fatty acids including ALA, EPA, DPA, and DHA. Yolk ALA, DPA, and DHA were increased linearly, yet EPA was reduced linearly, indicating that consumption of EUL (rich in ALA) enhanced the conversion of ALA into DHA by EPA and was deposited in yolk lipids, although the conversion efficiency remained limited. These findings were similar to previous reports on laying hens’ results [2,3,14]. ALA was the preferred substrate of Δ-6 desaturase enzyme (rate-limiting enzyme) compared to LA, so yolk n-3 PUFA content increased when substantial amounts of ALA were fed to poultry [6,38]. This might also explain the increased accumulation of n-3 PUFA without changes in n-6 PUFA and decreased n-6: n-3 ratio in yolk lipids from laying ducks fed EUL. The n-6: n-3 ratio is usually used to assess the nutritional quality of egg yolk fatty acids [6]. The lower the n-6: n-3 ratio, the more it benefited blood vessels and the more it tended to reduce the risk of cardiovascular strokes by improving lipid metabolism, inflammation, oxidative stress, and endothelial function [10]. In addition, the inclusion of EUL in the diets of laying ducks increased yolk oleic acid content yet decreased stearic acid content in a dose-dependent way, indicating that EUL enhanced conversion of stearic acid into oleic acid, with protective effects against oxidative stress and cytotoxicity [39]. Those data indicated that it was feasible to produce low n-6: n-3 PUFA eggs by enriching n-3 fatty acids by dietary EUL supplementation for laying ducks.

## 5. Conclusions

In summary, dietary EUL supplementation in laying ducks deposited phenols in the yolk, reduced yolk cholesterol contents, and lowered n-6: n-3 PUFA ratios by enriching n-3 fatty acids in yolks, without negative effects on performance, egg quality, or health status. Therefore, EUL rich in ALA can be utilized as an alternative source of ALA to produce duck eggs with a lower n-6: n-3 PUFA ratio and higher levels of total n-3 PUFA content.

## Figures and Tables

**Table 1:** Composition and nutrient levels of basal diets for laying ducks (air-dry basis).

Item	Dietary EUL (%)			
	0	1	2	4
Ingredient (%)				
Corn	57.21	57.40	57.44	58.00
Wheat bran	1.43	1.24	1.14	0.00
Rice hull powder	2.30	1.60	0.80	0.00
Soybean meal	27.20	26.90	26.76	26.06
Salt	0.30	0.30	0.30	0.30
CaHPO_4_	1.50	1.50	1.50	1.53
Limestone	7.44	7.44	7.44	7.44
Premix ^1^	1.00	1.00	1.00	1.00
DL-Methionine	0.12	0.12	0.12	0.14
Lysine	0.00	0.00	0.00	0.03
Soybean oil	1.50	1.50	1.50	1.50
EUL ^3^	0.00	1.00	2.00	4.00
Total	100.00	100.00	100.00	100.00
Calculated values				
AME (MJ/kg) ^2^	11.30	11.30	11.30	11.30
Crude protein (%)	17.15	17.18	17.28	17.20
Calcium	3.12	3.12	3.12	3.12
Available phosphorus	0.38	0.38	0.38	0.38
Methionine	0.39	0.39	0.39	0.40
Cysteine	0.30	0.30	0.30	0.29
Methionine + Cysteine	0.70	0.69	0.69	0.70
Lysine	0.87	0.86	0.86	0.86

^1^ Supplied per kilogram of total diet: Cu (CuSO_4_•5H_2_O), 10 mg; Fe (FeSO_4_•7H_2_O), 60 mg; Zn (ZnO), 60 mg; Mn (MnSO_4_•H_2_O), 100 mg; Se (NaSeO_3_), 0.3 mg; I (KI), 0.4 mg; choline chloride, 1500 mg; vitamin A (Retinol acetate), 10,000 IU; vitamin D_3_ (Cholcalciferol), 3000 IU; vitamin E (*DL-*α-tocopheryl acetate), 20 IU; vitamin K_3_ (menadione sodium bisulfate), 2.5 mg; thiamin (thiamin mononitrate), 2 mg; riboflavin, 15 mg; pyridoxine hydrochloride, 4 mg; cobalamin, 0.06 mg; calcium-*D*-pantothenate, 20 mg; nicotinic acid, 50 mg; folic acid, 1 mg; biotin, 0.2 mg. ^2^ The values are calculated according to the AME of feedstuffs for ducks [28]. ^3^ The AME of EUL is 0.736 MJ/kg.

**Table 2:** Effects of dietary EUL on production performance for laying ducks after 6 wk of feeding ^1^.

Item	Dietary EUL (%)				SEM	*p*-Value
	0	1	2	4		
Total egg mass (kg)	21.60	20.04	20.22	20.30	0.500	0.6971
Egg production (%)	65.38	64.00	61.88	62.75	1.643	0.8966
Average egg weight (g)	64.29	62.64	64.12	64.42	0.384	0.3374
Daily feed intake (g)	178.30	182.54	176.39	180.99	1.260	0.3254
Feed conversion ratio	4.36	4.65	4.52	4.50	0.111	0.8467
Daily egg mass (g/bird)	41.95	40.23	39.67	40.46	1.003	0.8833
Uncracked shell egg rate (%)	99.81	99.47	99.58	98.99	0.145	0.2376

^1^ Values are mean of 8 replicates of 12 laying ducks for the entire experiment.

**Table 3:** Effects of dietary EUL on plasma parameters of laying ducks after 6 wk of feeding ^1^.

Index	Dietary EUL (%)				SEM	*p*-Value
	0	1	2	4		
ALT (U/L)	34.92	41.33	41.33	47.92	1.891	0.1077
AST (U/L)	30.08	28.92	27.67	25.08	1.377	0.6400
TBA (μmol/L)	35.02	28.25	23.05	25.01	2.571	0.3929
Total protein (g/L)	46.15 ^b^	47.58 ^ba^	48.28 ^ba^	51.88 ^a^	0.734	0.0277
Albumin (g/L)	16.52	20.39	20.01	16.98	0.778	0.1708
Globulin (g/L)	21.52 ^b^	27.18 ^ba^	28.27 ^ba^	34.89 ^a^	1.631	0.0242
Glucose (mmol/L)	8.89	8.40	8.48	9.16	0.149	0.2361
Uric acid (mmol/L)	243.02	252.03	238.77	204.38	9.848	0.3561
Cholesterol (mmol/L)	4.73 ^a^	3.88 ^ba^	3.68 ^b^	3.82 ^ba^	0.150	0.0443
Triglyceride (mmol/L)	2.15	3.34	2.51	2.29	0.338	0.6282
HDL-C (mmol/L)	1.74 ^b^	1.80 ^b^	2.06 ^ba^	2.32 ^a^	0.070	0.0046
LDL-C (mmol/L)	2.18 ^a^	1.23 ^b^	1.19 ^b^	1.58 ^ba^	0.129	0.0142

ALT, alanine aminotransferase; AST, aspartate aminotransferase; TBA, total bile acid; HDL-C, high-density lipoprotein cholesterin; LDL-C, low-density lipoprotein cholesterin. ^1^ Values are mean of 8 replicates with 2 laying ducks being analyzed and averaged for each replicate. ^a,b^ Within a row, means without a common superscript differed (*p* < 0.05).

**Table 4:** Effect of dietary EUL on egg quality for laying ducks ^1^.

Item	Dietary EUL (%)				SEM	*p*-Value
	0	1	2	4		
Egg weight (g)	63.73	60.61	62.69	63.12	0.608	0.3028
Egg yolk weight (g)	19.86	18.63	19.48	19.49	0.301	0.5438
Egg yolk percentage (%)	31.19	30.92	31.06	30.88	0.387	0.9926
Egg shape index	1.31	1.30	1.30	1.31	0.006	0.6926
Eggshell thickness (mm)	0.43	0.43	0.43	0.44	0.002	0.8301
Albumen height (mm)	6.01	6.04	6.14	6.03	0.126	0.9835
Egg yolk color	4.88	5.44	5.88	5.75	0.161	0.1196
Haugh units	74.19	76.13	75.41	74.83	0.986	0.9214
Eggshell strength (N)	48.57	47.77	52.27	48.10	1.048	0.4121

^1^ Values are mean of 8 replicates with 2 eggs being analyzed and averaged for each replicate.

**Table 5:** Effect of dietary EUL on chemical composition of egg yolk for laying ducks (Fresh matter basis) ^1^.

Items	Dietary EUL (%)				SEM	*p*-Value
	0	1	2	4		
Dry matter (%)	50.03	49.32	50.30	50.71	0.256	0.2830
Crude protein (%)	14.97	14.76	14.98	14.72	0.077	0.5244
Total lipid (%)	27.86	27.28	28.00	28.59	0.199	0.1416
Total Phenolic Content (mg/g)	3.24 ^b^	3.30 ^ba^	3.42 ^ba^	3.66 ^a^	0.056	0.0313
Total Flavonoid Content (mg/g)	0.68	0.73	0.66	0.80	0.021	0.0902
Total triglyceride (mg/g)	48.10	51.70	46.18	48.21	0.803	0.0988
Total cholesterol (mg/g)	15.65 ^a^	14.04 ^ba^	13.50 ^b^	13.70 ^b^	0.309	0.0478

^1^ Values are mean of 8 replicates with 2 eggs being analyzed and averaged for each replicate. ^a,b^ Within a row, means without a common superscript differed (*p* < 0.05).

**Table 6:** Effect of EUL on fatty acids composition (%, *w*/*w*) in yolk after 6 wk of feeding ^1^.

Fatty Acids	Dietary EUL (%)				SEM	*p*-Value
	0	1	2	4		
C14:0	0.26	0.26	0.25	0.24	0.004	0.4972
C15:0	0.13	0.13	0.13	0.14	0.004	0.6592
C16:0	19.67 ^a^	19.51 ^a^	18.50 ^ba^	17.56 ^b^	0.263	0.0085
C17:0	0.33	0.33	0.36	0.38	0.011	0.3999
C18:0	8.02 ^a^	7.53 ^ba^	7.24 ^b^	7.20 ^b^	0.100	0.0069
C20:0	0.21	0.21	0.23	0.25	0.008	0.4033
C21:0	0.66	0.46	0.50	0.47	0.037	0.1897
C14:1 n-5	0.09	0.09	0.10	0.10	0.004	0.4403
C16:1 n-7	2.25	2.43	2.59	2.35	0.056	0.1701
C18:1 n-9	43.10 ^b^	44.63 ^ba^	45.04 ^a^	45.80 ^a^	0.297	0.0058
C18:1 n-7	2.36	2.37	2.36	2.38	0.031	0.9929
C20:1 n-9	0.65	0.67	0.71	0.69	0.022	0.8657
C18:2 n-6 (LA)	8.31	8.13	8.32	8.01	0.110	0.7219
C18:3 n-6	0.48	0.48	0.52	0.53	0.015	0.4767
C22:2 n-6	0.42	0.44	0.48	0.48	0.013	0.3128
C20:3 n-6	0.70	0.72	0.80	0.79	0.022	0.2534
C20:4 n-6 (AA)	7.42	6.93	6.88	7.33	0.147	0.4806
C22:4 n-6	0.64	0.68	0.72	0.73	0.020	0.4134
C22:5 n-6	2.22	2.09	2.19	2.21	0.060	0.8754
C18:3 n-3 (ALA)	0.42 ^b^	0.46 ^ba^	0.53 ^ba^	0.54 ^a^	0.016	0.0191
C22:5 n-3 (DPA)	0.36 ^b^	0.39 ^ba^	0.44 ^ba^	0.49 ^a^	0.016	0.02
C22:6 n-3 (DHA)	0.78 ^b^	0.83 ^b^	0.92 ^b^	1.15 ^a^	0.031	<0.0001
C20:5n-3 (EPA)	0.44 ^a^	0.24 ^b^	0.19 ^b^	0.20 ^b^	0.021	<0.0001
Total SFA	29.28 ^a^	28.42 ^ba^	27.22 ^bc^	26.23^c^	0.274	<0.0001
Total MUFA	48.45 ^b^	50.19 ^ba^	50.80 ^a^	51.33 ^a^	0.299	0.0013
Total PUFA	22.09	21.39	21.98	22.44	0.179	0.2154
n-3 PUFA	1.90 ^b^	1.92 ^b^	2.07 ^ba^	2.37 ^a^	0.053	0.0025
n-6 PUFA	20.20	19.47	19.91	20.08	0.157	0.3953
n-6: n-3	10.84 ^a^	10.32 ^a^	9.64 ^ba^	8.54 ^b^	0.249	0.0031
PUFA: SFA	0.76 ^b^	0.75 ^b^	0.81 ^ba^	0.86 ^a^	0.011	0.0004

LA, linoleic acid; AA, arachidonic acid; ALA, α-linolenic acid; DPA, docosapentaenoic acid; DHA, docosahexaenoic acid; EPA, eicosapentaenoic acid; SFA, saturated fatty acid; MUFA, monounsaturated fatty acid; PUFA, polyunsaturated fatty acid. ^1^ Values are mean of 8 replicates, with 2 eggs being analyzed and averaged for each replicate. ^a–c^ Within a row, means without a common superscript differed (*p* < 0.05).

## Data Availability

The data presented in this study are available on request from the corresponding author on reasonable request.

## Supplementary Materials

The following supporting information can be downloaded at: https://www.mdpi.com/article/10.3390/foods12020287/s1, Table S1: Chemical composition of the EUL sample (Dry matter basis).
