# Supplementary material for: Feeding Laying Ducks Eucommia ulmoides oliv. Leaves Increases the n-3 Fatty Acids Content and Decreases the n-6: n-3 PUFA Ratio in Egg Yolk without Affecting Laying Performance or Egg Quality
Source: Foods. 2023 Jan 8;12(2):287. doi: 10.3390/foods12020287 (PMC9857631; doi:10.3390/foods12020287)
Supplement: Supplementary file 1 [file foods-12-00287-s001.zip › foods-2114818-supplementary.pdf]

Table S1. Chemical composition of the EUL sample (Dry matter basis)

Items	Value
Moisture (%)	9.33
Crude protein (%)	17.18
Ether extract (%)	13.48
Crude fiber (%)	22.05
Ash (%)	7.97
Nitrogen free extract (%)	29.98
Calcium (%)	1.88
Phosphorus (%)	0.19
Total Phenolic Content %)	7.236
Total Flavonoid Content (%)	1.341
C8:0 (μg/g)	1.43
C14:0 (μg/g)	5.00
C15:0 (μg/g)	2.57
C16:0 (μg/g)	872.20
C17:0 (μg/g)	14.29
C18:0 (μg/g)	225.68
C20:0 (μg/g)	111.83
C21:0 (μg/g)	23.70
C22:0 (μg/g)	174.34
C23:0 (μg/g)	30.83
C22:1 (μg/g)	1.32
22:1 n-9 (μg/g)	9.35
C16:1 n-7 (μg/g)	16.69
C18:1 n=7 (μg/g)	53.15
C20:1 n-9 (μg/g)	24.48
C18:1 n=9 (μg/g)	564.15
C20:4 n-6 (AA) (μg/g)	6.94
C18:2 n-6 (LA) (μg/g)	515.97
C18:3 n-6 (μg/g)	1.30
C22:2 n-6 (μg/g)	3.93
C18:3 n-3(ALA) (μg/g)	1657.87
C20:3 n-3 (μg/g)	5.77
C22:2 (μg/g)	1.85
Nervonic Acid (μg/g)	2.92

LA, linoleic acid; AA, arachidonic acid; ALA, α-linolenic acid; EUL, Eucommia ulmoides oliver leaf powder.
